# The Neurostimulation of the Brain in Depression Trial: Protocol for a Randomized Controlled Trial of Transcranial Direct Current Stimulation in Treatment-Resistant Depression

**DOI:** 10.2196/22805

**Published:** 2021-03-17

**Authors:** Raheem Suleman, Benjamin V Tucker, Serdar M Dursun, Michael L Demas

**Affiliations:** 1 Department of Psychiatry Faculty of Medicine and Dentistry University of Alberta Edmonton, AB Canada; 2 Department of Linguistics Faculty of Arts University of Alberta Edmonton, AB Canada; 3 Grey Nuns Community Hospital Edmonton, AB Canada

**Keywords:** neuromodulation, neurostimulation, transcranial direct current stimulation, electrical stimulation therapy, psychiatric somatic therapies, depression, depressive disorder, major depressive disorder, depressive disorder, treatment resistant, randomized controlled trial, therapeutics, clinical trial protocol

## Abstract

**Background:**

Major depressive disorder (MDD) is the second highest cause of disability worldwide. Standard treatments for MDD include medicine and talk therapy; however, approximately 1 in 5 Canadians fail to respond to these approaches and must consider alternatives. Transcranial direct current stimulation (tDCS) is a safe, noninvasive method that uses electrical stimulation to change the activation pattern of different brain regions. By targeting those regions known to be affected in MDD, tDCS may be useful in ameliorating treatment-resistant depression.

**Objective:**

The objective of the Neurostimulation of the Brain in Depression trial is to compare the effectiveness of active versus sham tDCS in treating patients with ultraresistant MDD. The primary outcome will be the improvement in depressive symptoms, as measured by the change on the Mongtomery-Asberg Depression Rating Scale. Secondary outcomes will include changes in the Quick Inventory of Depressive Symptomatology Scale (subjective assessment), the World Health Organization Disability Assessment Schedule 2.0 (functional assessment), and the Screen for Cognitive Impairment in Psychiatry (cognitive assessment). Adverse events will be captured using the Young Mania Rating Scale; tDCS Adverse Events Questionnaire; Frequency, Intensity, and Burden of Side Effects Rating Scale; and Patient-Rated Inventory of Side Effects Scale. A parallel component of the study will involve assaying for baseline language function and the effect of treatment on language using an exploratory acoustic and semantic corpus analysis on recorded interviews. Participant accuracy and response latency on an auditory lexical decision task will also be evaluated.

**Methods:**

We will recruit inpatients and outpatients in the city of Edmonton, Alberta, and will deliver the study interventions at the Grey Nuns and University of Alberta Hospitals. Written informed consent will be obtained from all participants before enrollment. Eligible participants will be randomly assigned, in a double-blinded fashion, to receive active or sham tDCS, and they will continue receiving their usual pharmacotherapy and psychotherapy throughout the trial. In both groups, participants will receive 30 weekday stimulation sessions, each session being 30 minutes in length, with the anode over the left dorsolateral prefrontal cortex and the cathode over the right. Participants in the active group will be stimulated at 2 mA throughout, whereas the sham group will receive only a brief period of stimulation to mimic skin sensations felt in the active group. Measurements will be conducted at regular points throughout the trial and 30 days after trial completion.

**Results:**

The trial has been approved by the University of Alberta Research Ethics Board and is scheduled to commence in June 2021. The target sample size is 60 participants.

**Conclusions:**

This is a protocol for a multicenter, double-blinded, randomized controlled superiority trial comparing active versus sham tDCS in patients with treatment-resistant MDD.

**Trial Registration:**

ClinicalTrials.gov NCT04159012; http://clinicaltrials.gov/ct2/show/NCT04159012.

**International Registered Report Identifier (IRRID):**

PRR1-10.2196/22805

## Introduction

### Depression

As delineated in the *Diagnostic and Statistical Manual of Mental Disorders, Fifth Edition*, major depressive disorder (MDD) is characterized by the occurrence, and often recurrence, of major depressive episodes [1]. During these times, patients have low mood and/or anhedonia, in addition to at least four other criteria-specific symptoms encompassing emotional, cognitive, and somatic realms, for at least 2 weeks. MDD is a chronic, debilitating mental illness that exerts a profound effect on the quality of life of patients. Taken as a whole, depression is the second highest cause of disability both worldwide and in Canada, with an annual prevalence of 4.3% and a lifetime prevalence of 11.7% [2]. The resultant cost to Canada’s economy—from both direct and downstream effects—is a similarly high Can $32.3 billion (US $25.6 billion) annually, which is 2% of the nation’s gross domestic product [3]. Patients with depression have a 1.5-fold higher mortality rate and a 20-fold higher rate of completed suicide than their nondepressed counterparts [4,5].

Given its immense personal and societal impact, adequate treatment of depressive episodes is of utmost importance. From a physiological perspective, MDD likely arises from an imbalance in the activity of different neuronal circuits, leading to impairments in emotion regulation, social appraisal, cognitive functioning, and a variety of other mental domains [6]. All treatments, be they psychotherapeutic, pharmacological, or neuromodulatory in nature, function by restoring and ideally maintaining balanced neuronal function. Owing to their cost-effectiveness and ease of administration, medication and psychotherapy are first-line treatments for most subtypes of depression; however, 1 in 5 Canadians with MDD are deemed to have treatment-resistant depression that does not adequately respond to either of these modalities [7].

For these patients, the treatment of choice has traditionally consisted of electroconvulsive therapy (ECT), which was first pioneered in 1938 [8]. Although refinements to the procedure have been made since then, the general premise remains the same—by applying a brief, high-intensity electrical stimulus to the brain, one can induce a short seizure that treats depression through a variety of mechanisms [9]. Such treatments are given as a series of 6-16 sessions, in a highly monitored setting, and typically to inpatients in psychiatric facilities. Although effective, this method of neuromodulation has several limitations. From a health systems standpoint, it is expensive, time consuming, and resource intensive, requiring the services of a psychiatrist, anesthesiologist, and a variety of acute care allied health staff. As such, only specialized facilities routinely offer ECT, which can limit its utility in rural areas. Moreover, it can exert a deleterious effect on the cognitive function of patients during the course of treatment [10]. Even if patients do not experience significant side effects, the requirements of the treatment can impose a high iatrogenic burden. Many individuals are voluntarily hospitalized during their course of ECT, which can last a month or longer and can induce further disruption in their occupational and interpersonal lives. Others still choose not to even pursue ECT owing to their preconceived notions and the high stigma attached to it as a result of its portrayal in popular media [11,12].

### Transcranial Direct Current Stimulation

Options for treatment in individuals with refractory depression are limited, especially if they cannot pursue or have had no response to ECT. One promising method of neuromodulation that may be used in this scenario is transcranial direct current stimulation (tDCS). Unlike ECT, which uses a brief, high-intensity stimulus to induce seizures, tDCS consists of the application of a low-intensity current for longer periods (typically 20 to 30 minutes) through electrodes that are applied to the scalp. No seizures are generated, and as such, there is no need for general anesthesia or intensive monitoring; patients may even complete the treatment at home. The device itself, which is fairly simple from a mechanical perspective, is also cost-effective and can be purchased for less than Can $1000 (US $790). This compares favorably with ECT and with other neuromodulatory therapies such as repetitive transcranial magnetic stimulation (rTMS), which costs up to Can $75,000 (US $59,000) to purchase.

The mechanism by which tDCS exerts its effects is still under study. In part, it may induce long-term potentiation (LTP) and long-term depression (LTD), the mechanisms underlying neural plasticity, in targeted regions of the brain and modulate the activity of neural circuits without generating action potentials. Current flowing out of the anode may induce LTP and increase neuronal activity, whereas current flowing into the cathode may induce LTD and decrease brain activity [13]. Most studies of tDCS in the treatment of depression have applied anodal stimulation over the left dorsolateral prefrontal cortex (DLPFC) and cathodal stimulation over the right DLPFC—areas of the brain which are known to be hypometabolic and hypermetabolic, respectively, in patients with depression [14]. Due to its targeted nature, tDCS has not been shown to adversely affect cognition and may actually improve cognitive function over the course of treatment [15]. Electrodes can be accurately positioned by surface scalp measurement alone, and neuroimaging-guided localization is not required.

Previous studies have shown that tDCS is a safe and effective treatment for a variety of neuropsychiatric disorders, including schizophrenia, chronic pain, dementia, poststroke rehabilitation, and depression [16,17]. A meta-analysis by Brunoni et al [18], which examined 6 randomized control trials and a total of 289 patients with MDD, found that active tDCS was significantly superior to sham tDCS with respect to both response to treatment (odds ratio [OR] 2.44; number needed to treat [NNT]=7) and the induction of remission (OR 2.38; NNT=9) [18]. These results are similar to those observed for other therapies such as high-frequency rTMS, which has NNTs of 6 and 8 for response and remission, respectively [19].

These results suggest that tDCS may be an effective treatment for MDD; however, its role in treating more refractory cases remains unclear. The meta-analysis by Brunoni et al [18] found that the likelihood of response to tDCS was positively correlated with treatment duration and dose but negatively correlated with treatment resistance (defined as a failure to respond to 2 previous trials of antidepressants). This mirrors the findings seen with other neuromodulation techniques, including ECT and rTMS, and implies that patients with treatment resistance may need to be treated with higher-intensity protocols than their nonresistant counterparts. However, to date, only 3 studies have specifically examined tDCS in patients with treatment-resistant depression, and all have been limited by small sample sizes and relatively low-intensity protocols, among other methodological issues [20-22]. Unsurprisingly, these studies failed to demonstrate the effectiveness of tDCS, yet it remains unclear whether this is truly because of a lack of efficacy or because of issues with study design.

### Safety

In a meta-analysis examining over 33,200 sessions of tDCS, no serious adverse events were identified [23]. Common side effects of treatment that occur in up to 50% of patients include mild-to-moderate skin erythema, itchiness, tingling at electrode sites, or mild headache [20,24]. Less commonly observed side effects that have been reported include tinnitus, nervousness, light-headedness, blurred or brighter vision, reduced concentration, nausea, mild euphoria, fatigue, insomnia, or constriction when swallowing. When they occur, these side effects are generally transient and of low intensity.

In patients with bipolar depression, tDCS may rarely induce a manic or hypomanic switch. In a randomized control trial evaluating the use of tDCS in bipolar depression, treatment-emergent affective switches were observed in 15% of patients; however, these did not meet the criteria for hypomanic or manic episodes and did not require hospitalization or treatment discontinuation [25].

### Objectives

The main objective of this study is to compare the effectiveness of active versus sham tDCS in ameliorating depressive symptoms in participants with ultratreatment-resistant MDD. We have operationally defined ultraresistant MDD to include depression that has failed to remit despite the use of ketamine, ECT, or at least five previous antidepressant trials at clinically effective doses. The secondary objectives of the study are to compare the safety of active versus sham tDCS and their respective effects on the speech and language abilities of participants.

## Methods

### Trial Design

The Neurostimulation of the Brain in Depression (NESBID) trial is a pragmatic, randomized, parallel group, participant- and investigator-blinded multicenter superiority trial. Participants will be randomized in a 1:1 fashion between active and sham tDCS and will continue to receive their usual treatment, including pharmacological and psychological therapies, during the course of the trial.

### Eligibility Criteria

All participants will be required to sign an informed consent form before the final determination of their eligibility to enroll in the study. Participants will be eligible for inclusion if they:

- Are adults with a major depressive episode with a score greater than 34 (signifying severe depression) on the Montgomery-Åsberg Depression Rating Scale (MADRS)
- Have ultratreatment-resistant MDD (defined as failure to remit despite adequate trials with 5 antidepressants at clinically effective doses, failure to remit with ECT, or failure to remit with ketamine).

Participants will not be eligible for inclusion if they:

- Are currently diagnosed with psychosis, an addiction disorder (other than nicotine), borderline personality disorder, or antisocial personality disorder, as these conditions could interfere with adherence to the study protocol
- Are currently using a herbal compound or an agent known to modulate NMDA (N-methyl-D-aspartate) receptors, as these substances could interfere with the induction of LTP and thereby limit the effectiveness of tDCS
- Are pregnant, as tDCS has not been adequately studied in this population
- Have an electronic implant, cardiac dysrhythmia, seizure disorder, neurological disorder, or neurosurgical history, as the safety of electrical stimulation with tDCS cannot be assured given these comorbidities.

### Participant Recruitment and Randomization

Participants will be recruited from the inpatient population at the Grey Nuns and University of Alberta Hospitals in Edmonton, Alberta. In addition, outpatients in the city and surrounding areas will also be eligible for inclusion. Edmonton is a large and diverse Canadian city, with a metropolitan population of more than 1.3 million people. Participants may contact the study personnel directly or may be referred by their treating clinician; in the latter case, they must first give consent to disclose contact information to the study coordinators, who will then contact the participant and discuss the trial further. In cases where the treating clinician is also a study coordinator, the informed consent process will occur with a separate coordinator. Participants will be informed that they may withdraw their consent, without penalty, at any time and that this will not affect the care they receive from their treating clinician. Consent cannot be withdrawn after the conclusion of the protocol due to the blinding of participant-identifiable data.

Before randomization, participants will undergo a routine history and physical examination and complete the Edinburgh Handedness Inventory [26]. They will also be interviewed by a member of the research team using the Mini International Neuropsychiatric Interview [27]. This validated, structured tool facilitates the diagnosis of the 17 most common psychiatric pathologies and will provide confirmation that participant diagnoses fall within the acceptable purview of the inclusion and exclusion criteria. Participants will also complete a version of the Massachusetts General Hospital Antidepressant Treatment History Questionnaire, modified with the trade names of Canadian drugs [28]. To ensure that the information obtained is as accurate as possible, research personnel may also augment answers on this scale using historical medication information from a participant’s electronic health record. Finally, participants will have their baseline MADRS score recorded to ensure that it is higher than the threshold required for trial participation.

Once the study personnel confirm that participants satisfy the inclusion and exclusion criteria, they will be automatically assigned to a treatment arm using the Research Electronic Data Capture (REDCap) software, hosted at the University of Alberta [29,30]. Randomization will be performed in a permuted block fashion based on a sequence generated by the Robust Randomization App (RRApp) [31]. This sequence will not be known to the study team so as to preserve allocation concealment.

### Intervention and Control

In addition to continuing their usual pharmacological and psychological treatments, participants in both arms will receive 30 sessions of either sham or active tDCS, 5 days per week, for a total of 6 weeks, using a Sooma stimulator (Sooma Oy). This constitutes twice the number of treatment sessions as that used in the longest previously conducted study of tDCS in treatment-resistant depression [20]. For both groups, the anode will be positioned over the left DLPFC (position F3 on the 10-20 EEG system), and the cathode will be positioned over the right DLPFC (position F4). Electrodes will be positioned using the caps supplied by Sooma, with participant head circumference measured first to ensure that the proper-sized cap is applied. Each electrode pad will be saturated with 15 mL of normal saline before application.

Participants will be blinded to the group to which they have been assigned. Two stimulators labeled only as *A* and *B* will be set to provide either sham or active stimulation by an investigator not involved in participant interaction or data collection. In the sham group, the applied current will ramp up from 0.3 mA to 2 mA over 17 seconds and then ramp down to 0.3 mA over 17 seconds, where it will be maintained for the duration of the 30-minute session. Previous trials have demonstrated that this short period of active stimulation is an effective way of triggering scalp tingling at a total dose of tDCS believed to be below the threshold for inducing neuroplasticity changes [32,33]. Current must be maintained at 0.3 mA to allow for operation of the electrode-contact sensors; if these were deactivated entirely, blinding would be broken. In the active intervention group, the applied current will ramp up to 2 mA over 17 seconds and be maintained at that level for 30 minutes, before gradually ramping down.

Participants will be withdrawn from the study if they miss two consecutive treatments or more than five treatments in total, if they undergo a serious adverse event or clinical deterioration (which may include new-onset psychosis, severe suicidal ideation, or a manic or hypomanic switch), or if they or their treating psychiatrist wish them to be withdrawn.

In the unlikely event that a participant undergoes clinical deterioration or that their treating clinician requests their blind be broken, the sole unblinded investigator can be contacted to reveal the treatment code. If the treating clinician is also a study investigator and becomes unblinded as a result of this process, they will be required to recuse themselves from further data collection.

### Outcomes

The primary outcome will be the change on the MADRS, a 10-item, validated, observer-administered scale of depression severity, which has been extensively used in trials of depression treatment [34]. Investigators will use a structured interview guide when administering the MADRS to improve inter-rater reliability [35].

Secondary outcomes will include the change on the Quick Inventory of Depressive Symptomatology (QIDS-SR-16), a 16-item, participant-administered scale of depression severity, and the World Health Organization Disability Assessment Schedule (WHODAS 2.0), a 12-item measure of functional impairment [36,37]. Cognitive impacts of treatment will be assessed using the Screen for Cognitive Impairment in Psychiatry (SCIP) [38]. The SCIP has been well validated as a tool for measuring cognitive change in patients with depression and other psychiatric illnesses; is brief and relatively simple to perform; and includes a verbal list learning task, working memory test, verbal fluency test, delayed learning task, and visuomotor tracking test. Exploratory analyses will investigate the effect of treatment on language abilities by change in performance on an auditory lexical decision task, in which participants must differentiate real from fictitious words [39,40]. An exploratory acoustic and semantic corpus analysis will also be conducted on the recorded entrance and exit interviews.

Treatment-related manic or hypomanic switches will be captured using the Young Mania Rating Scale, an 11-item observer-administered scale of mania intensity [41]. Other treatment-related side effects will be measured using a scale derived from a systematic review of tDCS-related adverse events [42]. This scale has the benefit of asking participants to comment on the relatedness of their symptoms to their treatment and may help to distinguish between the likely side effects of tDCS and symptoms participants had before the intervention. It has also been used in other randomized control trials evaluating tDCS [43]. In addition, side effects that may be secondary to concurrent medication use will be captured using the Frequency, Intensity, and Burden of Side Effects Rating Scale and the Patient-Rated Inventory of Side Effects Scale [44,45]. Side effects captured through the use of the aforementioned scales will be summarized in an adverse events form, with additional documentation completed on any adverse events deemed to be serious in nature.

### Data Collection

Measurements for most scales will be conducted at the beginning of the trial (T0), after every 10 sessions (T10, T20, and T30), and 1 month after trial completion (T60; Table 1). Trained psychiatrists, psychiatric residents, and/or medical students will complete all assessments. Data will be collected directly using the REDCap electronic data capture tools.

Recorded entrance and exit interviews will be stored in an encrypted database and have identifying information stripped from them on a rolling basis. This database may then be analyzed in future studies.

Participants will perform the auditory lexical decision task on tablet devices using a previously developed app. They will first answer questions on their age, handedness, native languages, other spoken languages, age at which they began to learn English, English-speaking countries they have visited, and whether or not they grew up in western Canada. They will then hear a series of 200 stimuli and must mark whether they think each stimulus is a real or fictious word. There are a total of 2000 words split into 32 different lists. Participants will perform this task before commencing treatment (T0), after each stimulation session (T1-30), and 1 month after trial completion (T60).

There are 3 validated versions of the SCIP. To prevent participants from improving their scores by way of repeated exposure, version 1 will be administered at baseline, version 2 after the 30 treatments are delivered, and version 3 at the 1-month postcompletion assessment.

**Table 1:** Schedule of assessments.

Assessment			Timepoint								
			Enrollment and baseline^a^		Treatment period^b^						Follow-up^c^
			T0		T1-9; T11-19; T21-29		T10; T20		T30		T60
**Prerandomization**											
	Informed consent	✓^d^		—^e^		—		—		—	
	Screening of inclusion and exclusion criteria	✓		—		—		—		—	
	History and physical exam	✓		—		—		—		—	
	EHI^f^	✓		—		—		—		—	
	MINI^g^ (audio recorded)	✓		—		—		—		—	
	MGH-ATRQ^h^	✓		—		—		—		—	
	MADRS^i^ (audio recorded)	✓		—		—		✓		—	
**Postrandomization**											
	MADRS (not recorded)	—		—		✓		—		✓	
	QIDS-SR-16^j^	✓		—		✓		✓		✓	
	WHODAS^k^ 2.0	✓		—		✓		✓		✓	
	SCIP^l^	✓		—		✓		✓		✓	
	YMRS^m^	✓		—		✓		✓		✓	
	tDCS-AEQ^n^	✓		—		✓		✓		✓	
	FIBSER^o^	✓		—		✓		✓		✓	
	PRISE^p^	✓		—		✓		✓		✓	
	Auditory lexical decision task	✓		✓		✓		✓		✓	

^a^T0: baseline session.

^b^T1-30: treatment sessions 1 to 30.

^c^T60: follow-up one session, one month after trial completion.

^d^Assessment conducted.

^e^Assessment not conducted.

^f^EHI: Edinburgh Handedness Inventory.

^g^MINI: Mini International Neuropsychiatric Interview.

^h^MGH-ATRQ: Massachusetts General Hospital Antidepressant Treatment History Questionnaire.

^i^MADRS: Montgomery-Åsberg Depression Rating Scale.

^j^QIDS-SR-16: Quick Inventory of Depressive Symptomatology.

^k^WHODAS: World Health Organization Disability Assessment Schedule.

^l^SCIP: Screen for Cognitive Impairment in Psychiatry.

^m^YMRS: Young Mania Rating Scale.

^n^tDCS-AEQ: Transcranial Direct Current Stimulation Adverse Events Questionnaire.

^o^FIBSER: Frequency, Intensity, and Burden of Side Effects Rating Scale.

^p^PRISE: Patient-Rated Inventory of Side Effects Scale.

### Data Analysis and Sample Size Calculations

The primary and secondary efficacy outcomes will be analyzed using a mixed effects repeated measures ANOVA (analysis of variance), with the study group and time as fixed factors and participants as random factors, based on the intention to treat principle. Due to the wide variety of potential adverse events that can be captured, we will present this information as frequency data.

To detect a moderate effect size for the primary outcome, represented by a Cohen *f* of 0.25, with a power of 80% and significance ⍺=.05, we estimate that 60 participants will need to complete the protocol. This assumes a moderate correlation (*r*=0.3) between time points, using the power curves described in the textbook by Kirk [46]. As a study of this scope, in the ultraresistant MDD population, has not been performed before, we are unable to estimate the dropout rate. We plan to conduct an interim analysis after 30 participants complete the protocol. We will analyze the main effects of the treatment by presenting the significance of the time, treatment group, and interaction terms in the ANOVA. If there is a significant interaction, we will conduct posthoc comparisons between mean treatment and placebo outcome scores, at each time point, to characterize this effect further and will use the Tukey method to control the familywise error rate.

## Results

The trial has received approval from the University of Alberta Research Ethics Board and is registered on ClinicalTrials.gov (NCT04159012). We had planned to begin recruiting participants in 2019; however, all in-person research activities have since been suspended due to the COVID-19 pandemic and its physical-distancing implications. We anticipate that participant recruitment will begin in June 2021 at the Grey Nuns Hospital, with recruitment at the University of Alberta Hospital to begin thereafter. The trial is expected to run until June 2022.

## Discussion

### Study Overview

In this multicenter, double-blinded, randomized controlled trial, we aim to determine the effectiveness of tDCS in ameliorating ultratreatment-resistant MDD, operationally defined as depression that has failed to remit despite the previous use of at least five antidepressants at effective doses, ketamine, or ECT. In addition to their usual treatment, participants will be randomly assigned to receive either 30 weekday sessions of active (2 mA) or sham tDCS, with the anode over the left DLPFC and the cathode over the right DLPFC. We will regularly assess depression severity and functional impact using the MADRS, QIDS-SR-16, and WHODAS 2.0, throughout the trial and 1 month after study completion. We will assess cognitive changes using the SCIP. We will also regularly assess treatment-related side effects using validated scales.

As depression is known to affect speech [47-49], a parallel component of the study will examine how participant speech and auditory lexical decision changes throughout the trial period. Entrance and exit interviews will be recorded, transcribed (with identifying information removed), and analyzed by collaborators in the Department of Linguistics at the University of Alberta. Participant accuracy and response latency on the auditory lexical decision task will be similarly evaluated and compared.

### Strengths

The study has numerous strengths and will follow the CONSORT (Consolidated Standards of Reporting Trials) 2010 statement on design and reporting [50]. Using REDCap to randomly assign participants to treatment groups will ensure that allocation is truly concealed, documented, and unchangeable. To minimize the placebo effect, both participants and treating investigators will be blinded. Primary and secondary outcomes were chosen to capture a range of subjective, objective, and functional measurements of depression severity, and the regular use of adverse event scales will ensure that treatment side effects are also adequately captured. Measurements will also be taken at multiple points during and after the trial to better describe the overall course and persistence of any treatment-related effects.

This study is unique for several reasons. It is the first of its kind to examine a severely resistant MDD population, in which participants will have failed multiple previous treatments. The use of a relatively intensive tDCS regimen, both in terms of session duration and overall treatment course, is also unique among studies conducted with treatment-resistant patients. To our knowledge, this is also the first study of any therapeutic for MDD that includes detailed measures of language data and function. This may be useful in revealing the effect of tDCS on speech and in building a database of anonymized recordings for use in future analyses.

### Limitations

This study also has several practical limitations that may affect the results. With regard to treatment modality, delivering tDCS in a hospital setting will impose a minimal burden on inpatients, but outpatients will have to be functional enough to arrange for transport to the hospital on a daily basis. As such, there is a risk that the study population may miss outpatients with severe depression. The need for daily travel to the hospital can also pose problems with participant compliance. We may use home-based tDCS in future studies to mitigate this, although funding limitations prevented us from employing this approach in the NESBID trial. Second, our blinding procedure follows that used in most other studies of tDCS, but recent evidence suggests that this may not sufficiently mitigate the placebo effect. Turi et al [51] showed that healthy volunteers were able to distinguish the *fade-in, short stimulation, fade-out* method of sham stimulation, described above, from true active stimulation at a level significantly greater than chance. It is not yet known how generalizable these findings are to a population with depression, but there is a need for more research into alternative methods of blinding in tDCS studies.

### Conclusions

In conclusion, the NESBID trial is a pragmatic, multicenter, double-blinded study that seeks to compare the impact of active versus sham tDCS in treating ultraresistant depression. To our knowledge, this study is the first of its kind in this patient population and also the first to conjointly employ detailed measures of language function, which will also be used in future analyses. Given the immense health, economic, and societal impacts of MDD, new and effective treatments beyond pharmacotherapy and psychotherapy are needed, and tDCS may prove useful in this regard.

## Abbreviations

ANOVA: analysis of variance
DLPFC: dorsolateral prefrontal cortex
ECT: electroconvulsive therapy
LTD: long-term depression
LTP: long-term potentiation
MADRS: Montgomery-Åsberg Depression Rating Scale
MDD: major depressive disorder
NESBID: Neurostimulation of the Brain in Depression
NNT: number needed to treat
QIDS-SR-16: Quick Inventory of Depressive Symptomatology
rTMS: repetitive transcranial magnetic stimulation
SCIP: Screen for Cognitive Impairment in Psychiatry
tDCS: transcranial direct current stimulation
WHODAS: World Health Organization Disability Assessment Schedule
